# Use of Fitbit Devices in Physical Activity Intervention Studies Across the Life Course: Narrative Review

**DOI:** 10.2196/23411

**Published:** 2021-05-28

**Authors:** Ruth Gaelle St Fleur, Sara Mijares St George, Rafael Leite, Marissa Kobayashi, Yaray Agosto, Danielle E Jake-Schoffman

**Affiliations:** 1 Department of Public Health Sciences University of Miami Miller School of Medicine Miami, FL United States; 2 Department of Psychology University of Miami Coral Gables, FL United States; 3 Department of Health, Education, and Behavior University of Florida Gainesville, FL United States

**Keywords:** physical activity, Fitbit, eHealth, life course, mobile phone

## Abstract

**Background:**

Commercial off-the-shelf activity trackers (eg, Fitbit) allow users to self-monitor their daily physical activity (PA), including the number of steps, type of PA, amount of sleep, and other features. Fitbits have been used as both measurement and intervention tools. However, it is not clear how they are being incorporated into PA intervention studies, and their use in specific age groups across the life course is not well understood.

**Objective:**

This narrative review aims to characterize how PA intervention studies across the life course use Fitbit devices by synthesizing and summarizing information on device selection, intended use (intervention vs measurement tool), participant wear instructions, rates of adherence to device wear, strategies used to boost adherence, and the complementary use of other PA measures. This review provides intervention scientists with a synthesis of information that may inform future trials involving Fitbit devices.

**Methods:**

We conducted a search of the Fitabase Fitbit Research Library, a database of studies published between 2012 and 2018. Of the 682 studies available on the Fitabase research library, 60 interventions met the eligibility criteria and were included in this review. A supplemental search in PubMed resulted in the inclusion of 15 additional articles published between 2019 and 2020. A total of 75 articles were reviewed, which represented interventions conducted in childhood; adolescence; and early, middle, and older adulthood.

**Results:**

There was considerable heterogeneity in the use of Fitbit within and between developmental stages. Interventions for adults typically required longer wear periods, whereas studies on children and adolescents tended to have more limited device wear periods. Most studies used developmentally appropriate behavior change techniques and device wear instructions. Regardless of the developmental stage and intended Fitbit use (ie, measurement vs intervention tool), the most common strategies used to enhance wear time included sending participants reminders through texts or emails and asking participants to log their steps or synchronize their Fitbit data daily. The rates of adherence to the wear time criteria were reported using varying metrics. Most studies supplemented the use of Fitbit with additional objective or self-reported measures for PA.

**Conclusions:**

Overall, the heterogeneity in Fitbit use across PA intervention studies reflects its relative novelty in the field of research. As the use of monitoring devices continues to expand in PA research, the lack of uniformity in study protocols and metrics of reported measures represents a major issue for comparability purposes. There is a need for increased transparency in the prospective registration of PA intervention studies. Researchers need to provide a clear rationale for the use of several PA measures and specify the source of their main PA outcome and how additional measures will be used in the context of Fitbit-based interventions.

## Introduction

### Background

Insufficient physical activity (PA) in all stages of life, from early childhood to older adulthood, is a well-documented public health issue [1]. Between 2001 and 2016, although the levels of insufficient PA decreased marginally globally, high-income Western countries, such as the United States, reported a 5% increase in the prevalence of physical inactivity [2]. Insufficient PA is associated with increased risk for a variety of chronic diseases including cardiovascular disease, hypertension, and type 2 diabetes [3,4]. Although the current PA guidelines for Americans recommend at least 60 minutes per day of moderate- to vigorous-intensity PA for children and adolescents and 150 minutes per week of moderate-intensity PA for adults, more than 80% of youth and adults do not meet these guidelines [5].

Advances in 21st century technology have introduced the use of commercial off-the-shelf activity trackers (eg, Fitbit and Apple Watch) that allow users to self-monitor their daily PA. As one of the top 5 wearable companies based on shipment volume, Fitbit has produced some of the most popular fitness trackers that are currently available on the market [6]. These devices allow users to track their daily activities, including the number of steps, type of PA, and amount of sleep, among other features [7]. Fitbit released its first device in 2009 and its first wrist-worn tracker in 2012 [8]. The brand quickly gained popularity and saw a substantial increase in the use of activity trackers in a relatively short time. In 2014, Fitbit reported only 6.7 million active users compared with 29.6 million in 2019 [9]. In November 2019, Google announced its purchase of Fitbit for US $2.1 billion and publicly committed to accelerating innovation of these devices [7].

In the last decade, researchers have begun to take advantage of Fitbit’s public appeal, prominence, and relatively low cost compared with that of other commercial off-the-shelf activity trackers such as the Apple Watch, by incorporating these devices into their studies. This has been facilitated by Fitbit’s open application programming interface (API), which allows programmers to collect and store data across multiple devices [7]. Fitabase is an example of a company that capitalizes on Fitbit’s open API and works with researchers to collect, manage, and analyze data from participants’ Fitbit devices [10]. In addition to being a data management platform, Fitabase provides the general public with access to an extensive library containing hundreds of published studies, protocols, and methods papers that report their use of Fitbit devices [11]. As of January 7, 2021, 682 articles published between 2012 and 2018 were available on the Fitabase research library [11].

### Objectives

Early studies involving Fitbit focused on establishing its accuracy as an objective PA measurement tool, especially in comparison with existing gold standard measurement devices [12,13]. The first study using a Fitbit device to assess PA was published in 2012 and assessed its validity in measuring steps taken during self-paced and prescribed PA [14]. Overall, there have been mixed findings about the accuracy of Fitbit measurements, with some studies indicating step count accuracy 50% of the time compared with research-grade accelerometers [15] and others reporting high validity in step count measurements [16,17]. In addition to their ability to serve as a PA measurement tool, Fitbit devices are increasingly being used to support self-monitoring and goal setting as a way of promoting PA in intervention studies across the life course [18-21]. However, it is not clear how these commercially available devices are being incorporated into PA intervention studies. This gap severely hinders the creation of standardized procedures that operationalize Fitbit use in PA intervention studies (eg, wear time protocols, strategies to boost wear time, and analysis implications) [22]. An overview of the ways in which Fitbit devices can be used to measure or help achieve the desired intervention effects can further contribute to the evidence base. Notably, Fitbit devices have been used in PA interventions targeting children through older adults. However, differences in use protocols across age groups (eg, models and strategies to boost wear time) are not known. In this context, this narrative review aims to characterize how PA intervention studies across the life course use Fitbit in terms of device selection, intended use (intervention vs measurement tool), wear instructions, rates of adherence to device wear, strategies used to boost adherence, and potential use of additional PA measures. This review provides intervention scientists with a synthesis of information that may inform future trials involving Fitbit devices.

## Methods

### Search Strategy and Eligibility Criteria

Given that it serves as a repository of Fitbit-related studies, we first conducted a search of the Fitabase Fitbit Research Library [11]. As of January 7, 2021, the Fitabase research library included studies published between 2012 and 2018 and retrieved from PubMed, Google Scholar, the Association for Computing Machinery, JMIR, Science Direct, and IEEE. Approximately twice a week during this period, the Fitabase team conducted searches of those sources using the keyword *Fitbit*. The studies identified in the search were then put through a screening process wherein they were deemed eligible for inclusion in the library only if a Fitbit device was used as a key element of the study (ie, for measurement or intervention purposes) [11]. In the Fitabase library, we applied preexisting filters to limit eligible studies to those that were (1) intervention studies, (2) focused on and reported PA as a main study outcome, and (3) conducted in one of five developmental stages of interest (ie, childhood [9-12 years old], adolescence [13-17 years old], early adulthood [18-40 years old], middle adulthood [41-64 years old], or older adulthood [≥65 years old]). We excluded nonintervention studies, those that did not report a specific target population, and those that did not have full-text articles available. We also excluded intervention studies that used Fitbit devices exclusively to monitor sleep. To capture studies published between 2019 and 2020, we conducted a search of PubMed using the following string search: “(physical activity[Title/Abstract]) AND (Fitbit[Title/Abstract]) AND (intervention*[Title/Abstract]).” In addition to applying the inclusion and exclusion criteria specified earlier, we excluded protocol and review papers and qualitative studies.

### Data Collection

The first 2 authors created a standardized form for data extraction by using Microsoft Excel. The items on this form, which were all open-ended, captured (1) general study characteristics (ie, sample size, study design, and intervention description) and (2) Fitbit use (ie, model, wear time and adherence, strategies to boost wear time, and other measures of PA). After finalizing the form, the first author read all the eligible studies and extracted the relevant data. To enhance the reliability of the extracted information, 3 additional coders (RL, MK, and YA) subsequently read the articles and reviewed the extracted data. As part of our protocol, disagreements between authors were resolved through discussion, with the final decision being made by the senior author.

## Results

### Overview

**Figure 1:**
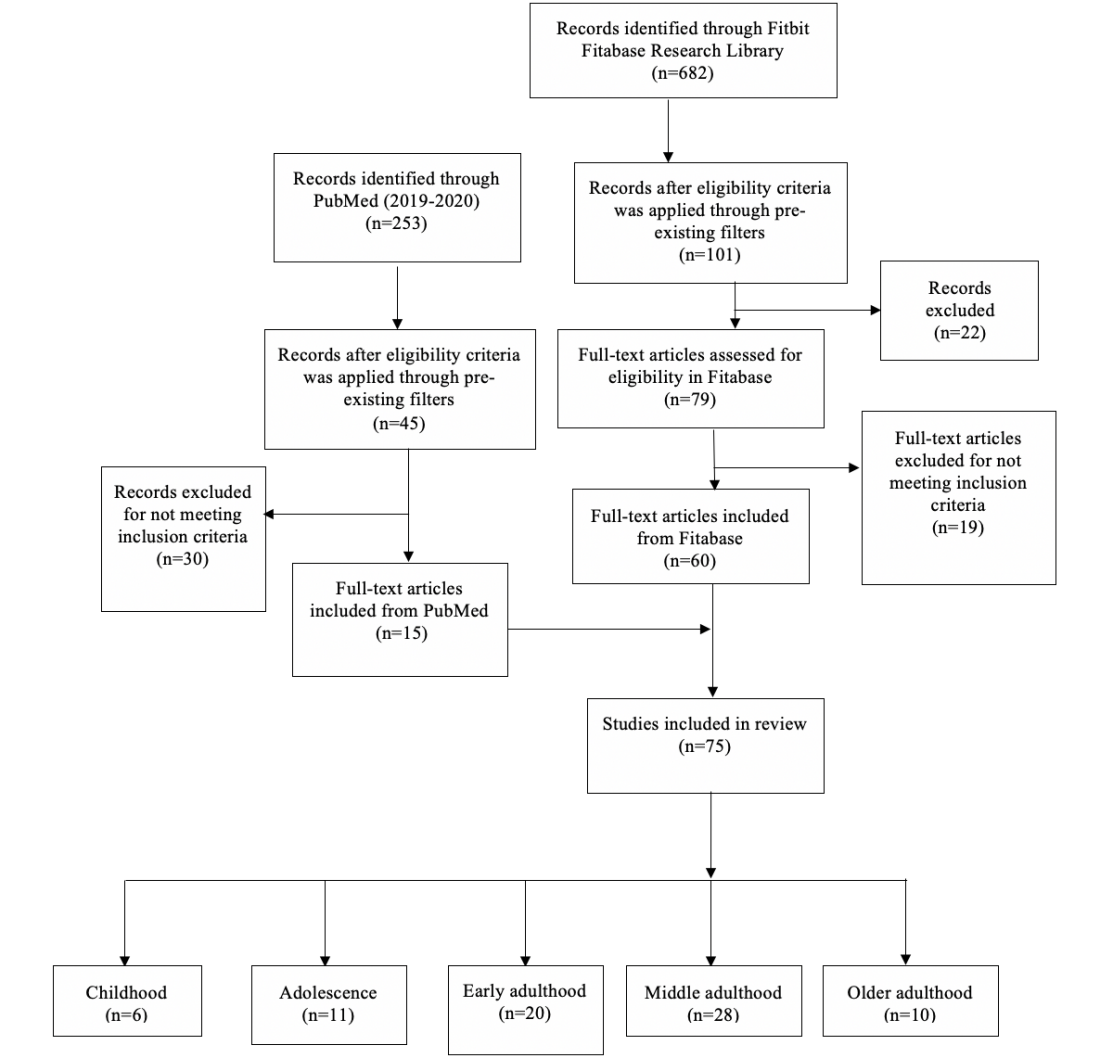
Study selection flow diagram.

Of the 682 studies available on the Fitabase Fitbit Research Library, 60 interventions met the eligibility criteria for this review. An additional 15 eligible studies resulting from the PubMed search were included. A total of 75 studies were reviewed (n=6 in childhood, n=11 in adolescence, n=20 in early adulthood, n=28 in middle adulthood, and n=10 in older adulthood). Figure 1 shows the flow diagram of the study. Tables 1 and 2 show the study characteristics and Fitbit use by developmental stage for included studies, organized by intended Fitbit use (ie, intervention vs measurement).

**Table 1:** General study characteristics.

Developmental stage				Study design and intervention description		Participant characteristics at baseline								
						Value, N		Age (years), mean (SD) or range		Female, %		Race or ethnicity		Weight status (eg, BMI, weight)
**Childhood**														
	**Intervention only**													
		Evans et al, 2017 [23]	Quasi-experimental design with 3 conditions: (1) Fitbit+intervention, (2) Fitbit only, and (3) control 6-week classroom-based intervention One session per week lasting 40 min and led by teachers and study staff Individual and group-level achievements BCTs^a^: goal setting, self-monitoring, and rewards		42		12.3 (0.3)		47^b^		NR^c^		42% overweight or obese	
		Mackintosh et al, 2016 [24]	Single-group pre-post design 4-week intervention with teams designing and completing week-long missions Teachers equipped with a guide and DVD outlining various missions BCTs: goal setting, self-monitoring, and rewards		30		10.1 (0.3)		40		NR		BMI: mean 19.9 (SD 4) kg/m^2^	
	**Measurement only**													
		Walther et al, 2018 [25]	Single-group pre-post design 12-week afterschool program with two 60-min sessions per week (24 total) 12 sessions focused on nutrition and increasing PA^d^ and 12 sessions taught safe food preparation while preparing simple, healthful recipes BCTs: shaping knowledge and self-monitoring		24		9.58 (NR)		83		30% White; 29% Black; 25% Hispanic; 16% Native American		NR	
	**Intervention and measurement**													
		Buchele Harris and Chen, 2018 [18]	Quasi-experimental design with 2 conditions: (1) PA engaging the brain+Fitbit challenge (PAEB-C) or (2) Fitbit only 4-week school-based intervention Participants in PAEB-C condition followed a 6-min video once a day BCTs: behavioral rehearsal and self-monitoring		116		10-11		49		60% reported race other than White, with 30% Black^b^		NR	
		Harris et al, 2018^b^ [26]	Quasi-experimental design with 2 conditions: (1) coordinated-bilateral PA intervention or (2) Fitbit only 4-week school-based intervention Repetitive coordinated-bilateral motor movements performed while following a 6-min video instruction once a day BCTs: behavioral rehearsal and self-monitoring		116		NR		50		60% reported race other than White, with 30% Black^b^		NR	
		Hayes and Van Camp, 2015 [27]	Single-group pre-post design 22 sessions of 20 min, 1 to 4 days per week on an elementary school playground during regularly scheduled, unstructured recess BCTs: self-monitoring		6		NR		100		NR		66% normal weight	
**Adolescence**														
	**Intervention only**													
		Chen et al, 2017 [28]	RCT^e^ with 2 conditions Phone-based 3-month intervention for adolescents who are overweight and obese 8 modules focused on lifestyle modification, weight management, nutrition, and stress BCTs: shaping knowledge and self-monitoring		40		14.9 (1.7)		42		90% Chinese American		BMI: mean 28.3 (SD 4.7) kg/m^2^	
		Gandrud et al, 2018 [29]	Parallel-group RCT with 2 conditions 6-month intervention using intensive remote therapy for pediatric patients with type 1 diabetes Content focused on recommendations for diabetes management, glucose control, and PA BCT: shaping knowledge and self-monitoring		117		12.7 (2.5)		54		NR		BMI z-score: mean 0.5 (SD 0.9)	
		Mendoza et al, 2017 [30]	Pilot RCT with 2 conditions 10-week intervention for adolescent and young adult survivors of cancer using a wearable device, mobile health app, and Facebook support group for reaching PA goals BCTs: shaping knowledge, self-monitoring, and social support		60		16.6 (1.5)		59		66% non-Hispanic White; 14% Hispanic; 7% non-Hispanic Black; 14% Other		NR	
	**Measurement only**													
		Haegele and Porretta, 2016 [31]	Single-group pre-post design Social cognitive theory–based PA intervention for adolescents with visual impairments 9 lessons delivered during PA classes that included curricular concepts, in-class activities, and homework BCTs: shaping knowledge, behavioral rehearsal, and self-monitoring		6		NR		NR		NR		NR	
		Meng et al, 2018 [32]	Quasi-experimental design 2-year intervention for soccer players delivered by coaches Content focused on addressing exercise, body image, and nutrition BCTs: shaping knowledge and self-monitoring		388		15.3 (1.1)		58		62% non-Latino; 38% Latino		BMI %: mean 62.8 (SD 25.0)	
		Walther et al, 2018 [25]	Pre-post study design 12-week intervention with fourth and fifth graders that focused on proper nutrition and safe food preparation techniques and promoted PA via interactive games BCTs: self-monitoring, shaping knowledge, and social support		30		9.58 (NR)		83		30% White; 29% Black or African American; 25% Hispanic; 16% Native American		NR	
	**Intervention and measurement**													
		Gaudet et al, 2017 [19]	Quasi-experimental crossover design 7-week classroom-based intervention to increase students’ PA BCTs: self-monitoring, self-regulation, and goal setting		46		13.0 (0.3)		52%		NR		NR	
		Pope et al, 2018 [33]	Multiphase mixed methods consisting of an RCT 12-week intervention for high school students where participants assigned to the game group were rewarded based on the number of daily steps taken BCTs: goal setting, self-monitoring, and rewards		105		17.0 (NR)		71		67% White; 16% Black; 12% Hispanic or Latino; 12% Asian; 5% Other		NR	
		Remmert et al, 2019 [34]	Quasi-experimental pilot study 12-week school-based ABT^f^ intervention to increase PA in adolescents with low activity Weekly sessions conducted by project coordinator consisted of acceptance-based behavioral counseling combined with preferred-intensity exercise for 30 min BCTs: behavioral counseling, behavioral practice, and self-monitoring		20		12.0 (0.0)		60		55% Latino; 25% non-Latino White; 20% Other		BMI: mean 21.7 (SD 3.6) kg/m^2^	
		Short et al, 2018 [35]	RCT with 2 conditions 48-week exercise intervention subdivided into 3 consecutive 16-week phases Tested how different incentive schemes influence exercise frequency and duration among youth Self-monitoring and rewards		77		14.0 (2.2)		NR		100% American Indian		BMI%: mean 98 (SD 3)	
		Van Woudenberg et al, 2018 [36]	RCT with 2 conditions 7-day classroom-based intervention that used a social network model to select and train influential adolescents (using smartphones) BCTs: social facilitation, behavior modeling, impression management, and self-persuasion		190		12.2 (0.5)		54		NR		NR	
**Early adulthood (18-40 years)**														
	**Intervention only**													
		Bang et al, 2017 [37]	Quasi-experimental design 6-week campus-based program with one session per week during lunch Participants walked together through the campus forest for approximately 40 min and received one lecture on stress management Encouraged to walk at least once per week at their leisure BCTs: self-monitoring, behavioral practice, and social support		99		24.8 (4.7)^b^		49^b^		NR		BMI: mean 21.9 (SD 2.9) kg/m^2b^	
		Baruth et al, 2019 [38]	Quasi-experimental pilot study with 2 conditions: (1) intervention and (2) control Weekly PA intervention for pregnant women until 35-week gestation BCTs: goal setting, behavior counseling, self-monitoring, and social support		45		28.4 (4.5)^b^		100		81.8% White^b^		BMI: mean 26.9 (SD 7.2) kg/m^2b^	
		Losina et al, 2017 [39]	Single condition feasibility study 6-month workplace program to increase PA among sedentary hospital employees through individual and team-based financial incentives BCTs: self-monitoring, goal setting, and rewards		292		38.0 (11.0)		83		62% White; 14% Black; 10% Asian; 7% Hispanic; 7% Other		32% normal weight; 30% overweight; 38% obese	
		Mahar et al, 2015 [40]	RCT with 2 conditions: (1) Fitbit and (2) no Fitbit 10-week intervention examined effects of movement technology on college students’ PA BCTs: self-monitoring		75		19.4 (1.2)		NR		NR		NR	
	**Measurement only**													
		Chen and Pu, 2014 [41]	RCT with 3 conditions: (1) competition, (2) cooperation or (3) hybrid One-week mobile app intervention to help promote exercise in pairs and earn badges based on performance BCTs: self-monitoring, social support, goal setting, and rewards		36		20-30		58		NR		2.8% underweight, 94% normal weight, 2.8% obese	
		Pagkalos et al, 2017 [42]	RCT with 2 conditions: (1) intervention and (2) control 5-week pilot study to monitor young adults’ exercise via a custom-built Facebook app for activity self-reporting BCTs: self-monitoring and social support		49		24.0 (7.0)		NR		NR		BMI: mean 22.5 (SD 3.0) kg/m^2^	
		Ptomey et al, 2018 [43]	RCT with 2 conditions: (1) exercise once a week and (2) exercise twice a week 12-week at-home intervention to increase MVPA^g^ using videoconferencing for groups of adults with Down syndrome BCTs: self-monitoring, behavioral practice, and social support		27		27.9 (7.1)		41		10% ethnic minorities		Group 1 BMI: mean 35.4 (SD 9.7) kg/m^2^; Group 2 BMI: mean 31.4 (SD 6.8) kg/m^2^	
		Walsh and Golbeck, 2014 [44]	Within-subject crossover study with 3 conditions: (1) social game using Fitbit steps as currency, (2) social interaction experience, and (3) control 30-day web-based intervention Participants in the social interaction could interact or communicate and share their PA levels with friends BCTs: self-monitoring, social support, and social comparison		74		37.7 (10.2)		59		NR		NR	
		Yoon et al, 2018 [45]	RCT with 2 conditions: (1) intervention and (2) control Observational PA data collected from participants over first 6 months Participants were sent a personalized email message about their activity to inform them of current PA levels and encourage increase in the last 6 months BCTs: self-monitoring and feedback on behavior		79		31.9 (9.6)		59		29.2% Hispanic		NR	
	**Intervention and measurement**													
		Choi, 2016 [46]	RCT with 2 conditions: (1) intervention mobile app+Fitbit and (2) Fitbit 12-week intervention with pregnant women between 10 and 20 weeks of gestation After an initial 30-min in-person intervention session, participants received daily message or video, encouragement, and activity diary through the app BCTs: self-monitoring, shaping knowledge, and written persuasion to boost self-efficacy		30		33.7 (2.6)		100		43% White; 40% Asian; 10% Hispanic; 7% Black		BMI (prepregnancy): mean 27.7 (SD 3.7) kg/m^2^	
		Chung et al, 2017 [47]	Single-group pre-post design stratified into 2 groups: (1) overweight or obese group and (2) healthy weight group 2-month intervention where participants received Twitter messages to encourage PA and healthy eating, photo-based messages, infographics, and website links related to healthy lifestyle behaviors BCTs: self-monitoring, shaping knowledge, and written persuasion to boost self-efficacy		12		19-20		67		50% White; 33% Black; 8% Asian; 8% American Indian		Group 1 BMI range: 25-35 kg/m^2^; Group 2 BMI range: 22-24.9 kg/m^2^	
		Gilmore et al, 2017 [48]	RCT for postpartum women with 2 conditions: (1) WIC^h^ standard care (WIC Moms) and (2) WIC standard care and personalized weight management via a smartphone (E-Moms) E-Moms group was given access to the SmartLoss SmartPhone app that included near real-time weight and activity monitoring, scheduled delivery of health information, and interventionist feedback BCTs: self-monitoring, feedback on behavior		35		26.0 (5.4)		100		74% African American		BMI: mean 32 (SD 3) kg/m^2^ (range 25.6-37.0 kg/m^2^)	
		Halliday et al, 2017 [49]	Pre-post study design A goal-focused exercise program that included weekly phone or face-to-face coaching to reinforce walking goals, as well as an optional 1-h supervised group walk on 2 occasions per week BCTs: self-monitoring, social support, behavioral practice, behavior counseling, goal setting		15		38.3 (6.4)		60		80% Caucasian		BMI: mean 30.4 (SD 6.4) kg/m^2^	
		Florence et al, 2016 [50]	RCT with 3 conditions: (1) group 1 (Fitbit+modules), (2) group 2 (Fitbit+modules+a social media-based game), (3) control group with just educational modules 14-week intervention for first-year medical students where daily steps and sleep hours were monitored in groups 1 and 2 during weeks 1-8 From week 9, all 3 groups had access to Fitbit Flex and the game platform, and students’ daily steps and sleep time were monitored until week 14 by Fitbit Flex BCTs: self-monitoring and social support		300		18-19		58		NR		NR	
		Miragall et al, 2017 [51]	RCT with 3 conditions: (1) IMI^i^+PED condition (access to IMI and use of a pedometer), (2) IMI condition (access to IMI and use of a blinded pedometer), and (3) control condition (use of a blinded pedometer) 3-week IMI conducted with sedentary or low-active students to increase motivation and set individualized PA goals BCTs: self-monitoring, goal setting, and verbal persuasion about self-efficacy		76		22.2 (3.7)		86		NR		BMI: mean 21.7 (SD 3.2) kg/m^2^	
		Schrager et al, 2017 [52]	Pre-post cohort study 1-month intervention where emergency medicine residents were asked to wear a Fitbit to assess its effects on their PA levels BCTs: self-monitoring		30		Median age: 28		47		NR		NR	
		Thorndike et al, 2014 [53]	2-phase intervention: phase 1 was a 6-week RCT and phase 2 was a 6-week nonrandomized team steps competition 12-week intervention that provided medical residents with free access to a fitness center, weekly one-hour personal training sessions, and up to 2 individual appointments with a Be Fit staff nutritionist BCTs: self-monitoring and shaping knowledge		108		29 (23-37)		54		66% White		BMI: mean 24.1 (range 17.8-35.6) kg/m^2^	
		Washington et al, 2014 [54]	Pre-post study design 3-week intervention in which participants won prizes for wearing their Fitbit and meeting experimenter-determined step criteria BCTs: self-monitoring, goal setting, and rewards		13		18-26		67		NR		NR	
		West et al, 2016 [55]	Quasi-experimental study design 9-week intervention where undergraduate students were assigned to either (1) a behavioral weight gain prevention intervention (healthy weight) or (2) an HPV^j^ awareness intervention 8 lessons on behavioral strategies to maintain weight and avoid obesity were delivered via electronic newsletters and Facebook postings BCTs: self-monitoring and shaping knowledge		58		21.6 (2.2)		81		90% White		BMI: mean 24.0 (SD 5.1) kg/m^2^	
		Zhang and Jemmott, 2019 [56]	Pilot RCT with 2 conditions: (1) intervention and (2) control 3-month intervention in small groups with mobile app to track group’s PA data and engage with others BCTs: self-monitoring, social support, and social comparison		91		26.8 (5.1)		100		100% African American		BMI: mean 31.6 (SD 8.2) kg/m^2^	
**Middle adulthood (41-64 years)**														
	**Intervention only**													
		Amorim et al, 2019 [57]	Pilot RCT with 2 conditions: (1) intervention and (2) control 6-month intervention with PA booklet, health coaching sessions, app, and Fitbit BCTs: self-monitoring, behavioral counseling, and shaping knowledge		68		58.4 (13.4)		50		NR		BMI: mean 28 (SD 5.5) kg/m^2^	
		Butryn et al, 2014 [58]	Single-group pre-post design 6 months group-based intervention with a web platform component to facilitate social connectivity BCTs: self-monitoring and social support		36		54 (7.18)		100		62% Caucasian		BMI: mean 32.7 (SD 7.32) kg/m^2^	
		Cadmus-Bertram al et, 2015 [59]	RCT with 2 conditions: (1) intervention (2) comparison (standard pedometer only) 16-week web-based self-monitoring intervention for inactive, postmenopausal women Content combined self-monitoring with self-regulatory skills, such as goal setting and frequent feedback BCTs: self-monitoring, knowledge shaping, self-regulation, goal setting, and feedback		51		60.0 (7.1)		100		92% non-Hispanic White^b^		BMI: mean 29.2 (SD 3.5) kg/m^2^	
		Cadmus-Bertram et al, 2019 [60]	Pilot RCT with 2 conditions: (1) intervention and (2) comparison 12-week multi-component intervention for cancer survivors and support partners with Fitbit linked to electronic health records BCTs: self-monitoring and social support		50		54.4 (11.2)		96		94% non-Hispanic White; 2% Hispanic; 2% Black; 2% Multiracial		BMI: mean 32.2 (SD 7.4) kg/m^2^	
		Dean et al, 2018 [20]	Quasi-experimental pilot study 8 weekly small group sessions Each 90-min session had a group discussion and an exercise component BCTs: self-monitoring, knowledge shaping, and social support		40		46.9 (9.8)		0		100% African American		67% obese	
		Duncan et al, 2020 [61]	RCT with 3 conditions: (1) enhanced, (2) traditional, and (3) control 6-month intervention for adults with overweight or obesity delivered via the app with educational content, dietary consultation, Fitbit, and scales Enhanced group received additional sleep intervention content via the app BCTs: self-monitoring, knowledge shaping, goal setting, and behavioral counseling		116		44.5 (10.5)		70.7		NR		BMI: mean 31.7 (SD 3.9) kg/m^2^	
		Ellingson et al, 2019 [62]	Randomized feasibility trial with 2 conditions: (1) intervention with Fitbit and (2) Fitbit only 12-week intervention with motivational interviewing, habit education, and Fitbit BCTs: self-monitoring and verbal persuasion to boost self-efficacy		91		41.7 (9.3)		53		79% White		BMI: mean 29.6 (SD 6.3) kg/m^2^	
		Kandula et al, 2017 [63]	16-week community-based, pre-post intervention Twice weekly group exercise classes, Fitbit Zip and web-based platform, goal setting, and classes on healthy eating BCTs: self-monitoring, social support, goal setting, and knowledge shaping		30		40 (5)		100		100% South Asian		BMI: mean 30 (SD 3) kg/m^2^	
		Ross and Wing, 2016 [64]	Randomized pilot trial with 3 conditions: (1) tech, (2) tech+phone, and (3) self-monitoring 6-month intervention with one group receiving self-monitoring tools (eg, booklets or scale) Tech group received Fitbit and tracked caloric intake through Fitbit app Tech+phone group received same materials along with 14 calls regarding behavioral weight loss techniques BCTs: self-monitoring, behavioral counseling, and knowledge shaping		80		51.1 (11.7)		86		84% Non-Hispanic White		BMI: mean 33 (SD 3.4) kg/m^2^	
		Singh et al, 2020 [65]	RCT with 2 conditions: (1) PA counseling, (2) PA counseling and Fitbit 12-week intervention for women with breast cancer that included a PA counseling session with exercise physiologist and educational booklet BCTs: self-monitoring, behavioral counseling, and knowledge shaping		52		Group 1: 52.8 (9.5); Group 2: 49.5 (8.6)		100		NR		Group 1: BMI: mean 28.5 (SD 5.2) kg/m^2^; Group 2: BMI: mean 28.7 (SD 6) kg/m^2^	
		Van Blarigan et al, 2019 [66]	Pilot RCT with 2 conditions: (1) intervention and (2) control 12-week intervention for cancer survivors with daily text messaging BCTs: self-monitoring and cues		42		54 (11)		59		73% White, 12% Asian, 12% Native American or other, 2% Black		BMI: mean 28.4 (SD 5.9) kg/m^2^	
	**Measurement only**													
		Patel et al, 2017 [67]	12-week family-based RCT intervention On the basis of behavioral economics and gamification principles, the intervention used points and levels (bronze, silver, gold, and platinum) to encourage families to change their behavior and increase their PA levels BCTs: self-monitoring, rewards, and social support		200		55.4 (NR)		56		100% Caucasian		BMI: mean 27.2 (SD 5.1) kg/m^2^^b^	
		Robinson et al, 2019 [68]	Pilot RCT with 2 conditions: (1) intervention and (2) control 5-week study using implementation intentions to establish PA habits using personalized materials BCTs: self-monitoring and knowledge shaping		63		49.4 (8.3)		72.6		NR		NR	
		Schumacher et al, 2017 [69]	Single-group pre-post trial study Partner-based PA program for women examining PA lapses, cognitive-affective responses to lapses, and the role of social support in PA BCTs: self-monitoring and social support		20		50 (7.2)		100		95% Caucasian		BMI: mean 30.9 (SD 8.9) kg/m^2^	
	**Intervention and measurement**													
		Adams et al, 2017 [70]	2×2 factorial, 4-month RCT with goal setting (adaptive vs static goals) and rewards (immediate vs delayed) WalkIT trial delivered intervention components by SMS text messages on a daily basis with prompt-to-action messages (eg, tips, questions, or motivational or inspirational messages) BCTs: self-monitoring, goal setting, shaping knowledge, persuasion to boost self-efficacy, and cues		96		41 (9.5)		77		81.3% Caucasian		BMI: mean 34.1 (SD 6.18) kg/m^2^	
		Arigo, 2015 [71]	Single-group pre-post design 4-week web-based intervention in pairs Participants have access to web-based modules and worksheets guiding them through seeking support and setting weekly PA goals BCTs: self-monitoring, social support, and goal setting		12		46 (13.1)		100		75% Caucasian		BMI: mean 32.6 (SD 5.7) kg/m^2^	
		Arigo et al, 2015b [72]	Single-group pre-post design 6-week program predominantly web-based with a single face-to-face session introducing PA promotion skills Participants were encouraged to communicate with their PA dyad partner and other participants BCTs: self-monitoring, goal setting, and social support		20		50 (7.2)		100		90% Caucasian		BMI: mean 30.9 (SD 8.9) kg/m^2^	
		Finkelstein et al, 2015 [73]	Randomized crossover design with 2 conditions: (1) message-on and (2) message-off 4-week web-based intervention targeted inactivity level with tailored text messages about sedentary time BCTs: self-monitoring and cues		27		52 (12.0)		100		47% White; 47% African American		BMI: mean 37.0 (SD 6.0) kg/m^2^	
		Fukuoka et al, 2018 [74]	Single-group pre-post trial, uncontrolled pilot study 8-week weight loss program for Latino adults at risk for type 2 diabetes Participants were provided with 2 in-person counseling sessions, Fitbit, use of the Fitbit app, and a Facebook group and were asked to track diet daily and weight twice per week BCTs: self-monitoring, behavioral practice, and social support		54		45.3 (10.8)		68.5		100% Latino		BMI: mean 31.4 (SD 4.1) kg/m^2^	
		Gell et al, 2020 [75]	Pilot RCT with 2 conditions: (1) intervention and (2) control with Fitbit 8-week intervention for cancer survivors with health coaching, text messaging, and Fitbit BCTs: self-monitoring, behavioral counseling, and cues		59		61.4 (9)		81		98.5% non-Hispanic White, 1.2% Black or Hispanic		BMI: mean 30.4 (SD 7) kg/m^2^	
		Gremaud et al, 2018 [76]	10-week RCT intervention comparing 2 arms: (1) Fitbit only and (2) Fitbit+MapTrek MapTrek, mobile phone–based walking game leverages Fitbit to track users’ PA and motivate users to engage in virtual walking races in numerous places around the globe BCTs: self-monitoring and feedback		146		40.6 (11.7)^b^		79.2^b^		91.7% Caucasian^b^		BMI: mean 29.9 (SD 6.6) kg/m^2^^b^	
		Grossman et al, 2017 [77]	16-week behavioral pre-post pilot program for postmenopausal women The program consisted of face-to-face group meetings every month, weekly weigh-ins, electronic check-ins, calorie-restricted diet, and high-intensity interval training BCTs: self-monitoring, social support, and behavioral practice		11		59.53 (11.7)		100		NR		BMI: mean 32 (SD 2.53) kg/m^2^	
		Linke et al, 2019 [78]	One-arm pilot study 12-week intervention for veterans recovering from substance use disorder that included psychoeducation classes, gym membership, and Fitbit BCTs: self-monitoring, social support, and knowledge shaping		15		45 (9.7)		13		60% non-Hispanic White, 27% Black, 13% Hispanic		NR	
		Meints et al, 2019 [79]	Prospective cohort study 26-week intervention for hospital employees to increase PA with financial incentives Groups of 3 were formed and financial incentives were given if team members met goals BCTs: self-monitoring, social support, rewards, and goal setting		225		Black participants: 43 (10); White participants: 39 (12)		84		81% White; 19% Black		Black participants: 84% had overweight or obesity; White participants: 68% had overweight or obesity	
		Painter et al, 2017 [80]	Retrospective analyses of 6 weight loss programs Participants were taught self-management strategies and were given a Fitbit, Wi-Fi-enabled scale, digital food and exercise log, and access to expert coach via electronic messages BCTs: self-monitoring and behavioral counseling		2113		44.54 (10.72)		59		NR		BMI: mean 33.8 (SD 6.8) kg/m^2^	
		Reed et al, 2019 [81]	Randomized repeated-measures study with 2 conditions: (1) intervention and (2) control 12-week intervention with self-regulatory PA strategies, weekly text messaging, and Fitbit BCTs: self-monitoring, self-regulation, and cues		59		48 (NR)		79.3^b^		93.2% White^b^		Weight: mean 92.47 (SD 22.8) kg^b^	
		Wang et al, 2015 [82]	RCT with 2 conditions: (1) text messaging+Fitbit and (2) Fitbit only 6-week intervention for adults with overweight and obesity receiving Fitbit and 3 daily SMS text messages prompting PA BCTs: self-monitoring and cues		67		48.2 (11.7)		91		67% White; 16% Hispanic; 4% African American; 3% Asian; 3% Other		BMI: mean 31 (SD 3.7) kg/m^2^	
		Willis et al, 2017 [83]	Randomized feasibility study with 2 conditions: (1) web-based social network delivery and (2) conference call delivery 6-month weight loss intervention Web-based social network condition had 24 weekly web-based modules led by health educators Conference call condition consisted of 24 weekly 60-min phone conferences BCTs: self-monitoring, social support, and knowledge shaping		70		47 (12.4)		84		24.3% minorities		BMI: mean 36.2 (SD 4) kg/m^2^	
**Older adulthood (≥65 years)**														
	**Intervention only**													
		Ashe et al, 2015 [84]	Randomized pilot trial with 2 conditions: (1) intervention and (2) comparison (educational sessions) 6-month intervention to increase PA through social support, group-based education, and individualized PA prescription BCTs: self-monitoring, knowledge shaping, and social support		25		64.1 (4.6)		100		NR		BMI: mean 26.9 (SD 6.8) kg/m^2b^	
		Christiansen et al, 2020 [85]	RCT with 2 conditions: (1) intervention and (2) control 6-month intervention for total knee replacement patients that included physical therapy, Fitbit, step goals, and monthly call with physical therapist BCTs: self-monitoring, goal setting, and behavioral counseling		43		67 (7)		53.4		91% White		BMI: mean 31.5 (SD 5.9) kg/m^2^	
		Kenfield et al, 2019 [86]	Pilot RCT with 2 conditions: (1) intervention and (2) control 12-week intervention for men with prostate cancer that included personalized health recommendations, Fitbit, study website, and text messages BCTs: self-monitoring, knowledge shaping and cues		76		65 (NR)		0		84% White		41% overweight, 35% with obesity	
		Thompson et al, 2014 [21]	Randomized controlled crossover trial with 2 conditions: (1) immediate intervention and (2) delayed intervention 48-week total: 24-week intervention that combined accelerometers with exercise counseling and 24 weeks without intervention Content included materials on exercise, goal setting, and tracking PA BCTs: self-monitoring, goal setting, behavioral counseling, and knowledge shaping		48		79.5 (7.0)		81		NR		Weight: mean 75.7 (SD 13.4) kg^b^	
	**Measurement only**													
		Rossi et al, 2018 [87]	Single-group study (survey and qualitative interviews) Participants wore Fitbit for 30 days to evaluate acceptability and validity of the device in diverse cancer survivors BCTs: self-monitoring		25		62 (9)		100		36% non-Hispanic White; 36% Hispanic; 16% non-Hispanic Black; 12% Asian		BMI: mean 32 (SD 9) kg/m^2^	
		Schmidt et al, 2018 [88]	Single-group study Participants wore Fitbit for 14 consecutive days and social cognitive factors, health issues, and views on aging were assessed BCTs: self-monitoring		40		66.3 (3.19)		62.5		NR		BMI: mean 25.19 (SD 3.52) kg/m^2^	
		Streber et al, 2017 [89]	RCT with 2 conditions: (1) intervention and (2) control with weekly gymnastics or cognitive training 12-week intervention with 90-min weekly sessions including PA program with social and cognitive activities and PA coaching program BCTs: self-monitoring, social support, knowledge shaping, and behavioral counseling		87		76 (9.2)		78		NR		NR	
	**Intervention and measurement**													
		Harkins et al, 2017 [90]	RCT with 4 conditions: (1) financial incentive, (2) social goals, (3) combined, and (4) control 16-week intervention to test use of financial incentives and donations on PA increase with 4-week follow-up that included pedometer, goal setting, and weekly feedback on goal attainment BCTs: self-monitoring, rewards, goal setting, and feedback		94		80.3		74		98% Caucasian		NR	
		McMahon et al, 2017 [91]	2×2 randomized factorial experiment with 4 conditions receiving PA protocol and Fitbit: (1) interpersonal BCS^k^, (2) intrapersonal BCS, (3) interpersonal and intrapersonal BCS, and (4) control based on receipt of interpersonal and intrapersonal behavior change strategies 8-week intervention with weekly 90-min meetings with all conditions receiving PA protocol, Fitbit, and workbook BCTs: self-monitoring, knowledge shaping, and social support		102		79 (NR)		75		75% White; 25% Black		NR	
		Vidoni et al, 2016 [92]	Randomized crossover trial with 2 conditions: (1) immediate intervention and (2) delayed intervention 16-week trial divided into 8-week intervention and 8-week baseline or maintenance phase data collection Intervention included the use of a Fitbit device and PA prescription BCTs: self-monitoring and goal setting		30		With cognitive impairment: 72.3 (5.2); without cognitive impairment: 69.6 (5.8)		With cognitive impairment: 43; without cognitive impairment: 89		With cognitive impairment: 90% White; 10% African- American; without cognitive impairment: 100% White		BMI (with cognitive impairment): mean 29.4 (SD 3.8) kg/m^2^; BMI (without cognitive impairment): mean 27.8 (SD 4.3) kg/m^2^	

^a^BCT: behavior change technique.

^b^Only intervention condition data reported.

^c^NR: not reported.

^d^PA: physical activity.

^e^RCT: randomized controlled trial.

^f^ABT: acceptance-based therapy.

^g^MVPA: moderate-to-vigorous physical activity.

^h^WIC: women, infants, and children.

^i^IMI: internet-based motivational intervention.

^j^HPV: human papillomavirus.

^k^BCS: behavior change strategy.

**Table 2:** Description of Fitbit use.

Study				Fitbit		Wear instructions		Fitbit use adherence							Fitbit used in comparison group?			Other PA^a^ measures
								Minimum wear time criteria		Rate		Strategies to boost adherence						
**Childhood**																		
	**Intervention only**																	
		Evans et al, 2017 [23]	Zip (phase 1) and charge (phase 2)		Phase 1: all waking hours 7 days/week; phase 2: 24 h, 7 days/week		Minimum of 8 h/day		Days participants were adherent in phase 1: 64.8%; days participants were adherent in phase 2: 73.4%^b^		After-session meetings with study staff to sync their Fitbit data		Yes; same for Fitbit-only comparison condition; no device for control group			Sensewear, Armband Mini, and Jawbone		
		Mackintosh et al, 2016 [24]	Zip		Duration of intervention		Entire duration of session		100% adherence (with staff monitoring)		NR^c^		N/A^d^			Accelerometry		
	**Measurement only**																	
		Walther et al, 2018 [25]	Charge HR		24 h for 7 days, including one weekend		NR		NR		NR		N/A			Self-reporting		
	**Intervention and measurement**																	
		Buchele Harris and Chen, 2018 [18]	Charge HR		Daily; 5 school days/week for 4 weeks		Minimum of 14 h/day		Average loss of 1-day data per person per week		Log sheets record PA		No			NR		
		Harris et al, 2018^b^ [26]	Charge HR		Daily; 5 school days/week for 4 weeks		NR		NR		Devices were charged at the end of the week		Yes; same use			NR		
		Hayes and Van Camp, 2015 [27]	Classic		Duration of intervention recess session		Entire duration of 20-min recess session		100% adherence (with staff monitoring)		NR		N/A			Second Fitbit		
**Adolescence**																		
	**Intervention only**																	
		Chen et al, 2017 [28]	Flex		Daily for 3 months		NR		NR		Weekly text reminders and phone calls		No			Self-reporting of PA using the California Health Interview Survey		
		Gandrud et al, 2018 [29]	NR		NR		NR		NR		Weekly reminders sent to upload data		Yes			NR		
		Mendoza et al, 2017 [30]	Flex		Daily for 10 weeks		Minimum of 500 steps/day		Days participants were adherent: 72%		Text reminders sent every other day to encourage PA goals		No			Accelerometry		
	**Measurement only**																	
		Haegele and Porretta, 2016 [31]	Zip		NR		NR		NR		NR		N/A			NR		
		Meng et al, 2018 [32]	Zip		7 days/week at baseline and post measures		Minimum of 8 h/day		NR		Daily texts or email reminders		Yes; device masked with duct-tape			NR		
		Walther et al, 2018 [25]	Charge		Wear on the 2nd and 10th week of the intervention for 7 days, including 1 weekend		24 h		NR		NR		N/A			Self-reported days of 60-min PA		
	**Intervention and measurement**																	
		Gaudet et al, 2017 [19]	Charge HR		Daily for 7 weeks		Minimum of 10 h/day		Median participant adherent 67% of intervention days		NR		Yes			Accelerometry and self-reporting		
		Pope et al, 2018 [33]	Flex		Daily for 12 weeks		NR		15% of students wore their Fitbit for <10 days; 36% never wore their Fitbit		Weekly lottery to win US $10 Amazon gift cards, weekly email reminders, and in-person troubleshooting at school once a week		Yes			NR		
		Remmert et al, 2019 [34]	Flex 2		Daily for 12 weeks		NR		Average number of days of valid Fitbit wear: 78 (out of 84 days)^b^		NR		Yes			Accelerometry		
		Short et al, 2018 [35]	Zip		Daily for 7 days		NR		NR		NR		Yes			NR		
		Van Woudenberg et al, 2018 [36]	Flex		Daily for 7 days		Minimum of 1000 steps/day		Days participants were adherent: 73.4%		NR		Yes			NR		
**Early adulthood (18-40 years)**																		
	**Intervention only**																	
		Bang et al, 2017 [37]	Zip		NR		NR		NR		NR		No			IPAQ^e^		
		Baruth et al, 2019 [38]	Charge		Daily for duration of intervention		Minimum one day per week		Fitbit worn on 93% of intervention weeks		NR		No			Accelerometry		
		Losina et al, 2017 [39]	Flex		Daily for duration of intervention		Minimum of 10 h/day		NR		NR		N/A			Self-reporting		
		Mahar et al, 2015 [40]	Flex		Daily for duration of intervention		NR		NR		NR		No			Self-reporting		
	**Measurement only**																	
		Chen and Pu, 2014 [41]	Ultra and One		Daily for 2 weeks		NR		NR		Daily reminder to share experience of wearing Fitbit		No			NR		
		Pagkalos et al, 2017 [42]	Zip		Daily for duration of intervention		NR		NR		NR		No			Self-reporting		
		Ptomey et al, 2018 [43]	Charge HR		During intervention sessions		NR		100% (with staff supervision)		NR		No			NR		
		Walsh and Golbeck, 2014 [44]	Classic		Daily for 10 days		NR		73% of participants were adherent		NR		Yes; same use			IPAQ		
		Yoon et al, 2018 [45]	Flex		Daily for duration of intervention		NR		Days participants were adherent: 66%		NR		Yes; same use			Self-reporting		
	**Intervention and measurement**																	
		Choi et al, 2016 [46]	Ultra		Daily for at least 10 h		Minimum of 1000 steps/day		Days participants were adherent: intervention: 78%; comparison: 80%		Participants entered steps into their daily activity diary		Yes; same use			Self-reporting		
		Chung et al, 2016 [47]	Zip		Daily for duration of intervention		NR		Days participants were adherent: overweight group: 99%; normal weight group: 78%		Study team sent Twitter message reminders		N/A			NR		
		Gilmore et al, 2017 [48]	Zip		Daily		NR		NR		NR		No			NR		
		Halliday et al, 2017 [49]	NR		Daily for duration of intervention		100 or more steps per day		50.5%-82.9% of participants adhered to wearing Fitbit on a weekly basis		Participants were invited to join a private group on the Fitbit website that allowed for data sharing		N/A			NR		
		Florence et al 2016 [50]	Flex		Daily for duration of intervention		NR		NR		NR		Yes; control group started Fitbit Flex on week 8			IPAQ		
		Miragall et al, 2017 [51]	One		Daily for duration of intervention		NR		N/A		N/A		Yes; blinded			NR		
		Schrager et al, 2017 [52]	Flex		Daily for duration of intervention		100 or more steps per day		Median number of eligible days where the participant recorded at least 100 steps was 27.5 (IQR 8)		Participants were given a 2-week acclimatization period to wear and use the device		N/A			Self-reporting of PA		
		Thorndike et al, 2014 [53]	Classic		Duration of intervention		500 or more steps/day		Percentage of worn days in each phase: 77% in phase 1 and 60% in phase 2		Weekly reminder emails to charge device and monetary incentives for high compliance rates		Yes; blinded			NR		
		Washington et al, 2014 [54]	Classic		Daily for duration of intervention		NR		2 subjects had missing Fitbit data		Participants earned opportunities to draw prizes and brought the device to the lab 3 times a week for charging and retrieving data		N/A			Self-reporting of PA		
		West et al, 2016 [55]	Zip and Aria		Daily for duration of intervention		NR		Students used their Fitbit for an average of 23.7 days (SD 15.2 days)		NR		No			NR		
		Zhang and Jemmott, 2019 [56]	Zip		Daily for duration of intervention		NR		16% of Fitbit data were missing during intervention period		Daily notifications to wear Fitbit and log PA		Yes; same use			NR		
**Middle adulthood (41-64 years)**																		
	**Intervention only**																	
		Amorim et al, 2019 [57]	NR		Daily		N/A		96% reported wearing every day or most days		NR		No			Accelerometry and IPAQ		
		Butryn et al, 2014 [58]	Flex		Daily for duration of intervention		NR		Participants wore 86% of days during intervention		Public display of PA data		N/A			GT3X+accelerometers		
		Cadmus-Bertram et al, 2015 [59]	One		Daily for duration of intervention		Minimum of 2000 steps/day		NR		NR		No			Accelerometry		
		Cadmus-Bertram et al, 2019 [60]	Charhe HR or Charge 2		Daily		N/A		NR		In-person instruction on Fitbit use		No			Accelerometry		
		Dean et al, 2018 [20]	Flex		Daily; duration of intervention		NR		Participants who were adherent to wear instructions: 70%		Participants received 3 text messages weekly		N/A			Community Health Activities Model Program for Seniors Questionnaire		
		Duncan et al, 2020 [61]	Alta		NR		NR		NR		NR		Yes, for both intervention groups; no, for control group			Accelerometry and Active Australia Survey		
		Ellingson et al, 2019 [62]	Charge		Use at participants’ discretion for duration of intervention		Minimum of 10 h/day		NR		Intervention group determined cues to remember to wear Fitbit and check data		Yes; same use			Accelerometry		
		Kandula et al, 2017 [63]	Zip		Daily		NR		NR		NR		N/A			Actigraph Accelerometer and self-reported questionnaire		
		Ross and Wing, 2016 [64]	Zip and Aria		Daily		NR		Days participants were adherent: Tech: 76%; Tech+phone: 86%		Fitbit sent weekly emails updating progress		Fitbit used in one comparison group but not the other (pedometer used)			NR		
		Singh et al, 2020 [65]	Charge		As desired to self-monitor and manage PA		NR		Average h worn: 17.3 h (SD 5.7 h) per 6.1 days (SD 0.8 days) per week		Basic instruction on using and setting up Fitbit		No			Accelerometry and Active Australia Survey		
		Van Blarigan et al, 2019 [66]	Flex		Daily		NR		Participants wore Fitbit for 88% of study days		N/A		No			Accelerometry		
	**Measurement only**																	
		Patel et al, 2017 [67]	Flex		Daily		At least 1000 steps/day		10.1% of missing observation days in intervention arm and 12.7% in control arm		NR		Yes			NR		
		Robinson et al, 2019 [68]	Zip		Daily during waking hours		NR		NR		Participants asked to sync Fitbit data daily		Yes; same use			NR		
		Schumacher et al, 2017 [69]	Flex		Daily		Minimum of 100 steps/day		97% adherent to wear time criteria		NR		N/A			NR		
	**Intervention and measurement**																	
		Adams et al, 2017 [70]	Zip		Daily during waking hours		NR		NR		Text step counts daily and random selection for monthly incentives for wearing their Fitbit regularly		Yes			IPAQ		
		Arigo, 2015 [71]	Flex		Daily; duration of intervention		NR		Days participants were adherent: 93%		Badges for achieving PA milestones; participants were advised to check step progress daily		N/A			NR		
		Arigo et al, 2015b [72]	Flex		Daily for duration of intervention		Defined as >100 steps in a day		Participants wore 97% of days during intervention		Instructions on device use, public display of steps data, and PA partner accountability		NA			NR		
		Finkelstein et al, 2015 [73]	One		Daily		NR		3 participants did not provide Fitbit data		Instructions and use of device before study for comfort and familiarity		Yes			Self-reporting		
		Fukuoka et al, 2018 [74]	Zip		Daily		Minimum of 8 h/day		NR		NR		N/A			IPAQ short version		
		Gell et al, 2020 [75]	One		Daily for duration of intervention		Minimum of 10 h/day		Average days participants were adherent: 6 days/week		NR		Yes; same use			Accelerometry		
		Gremaud et al, 2018 [76]	Zip		Daily during waking hours		NR		64.6% wear time in Fitbit arm with a 16.5% increase for Fitbit+Map Trek arm		Reminder system, which prompted each user to wear their Fitbit following nonwear days		Yes			NR		
		Grossman, et al 2017 [77]	Charge HR		Duration of intervention		NR		NR		NR		Yes			NR		
		Linke et al, 2019 [78]	Charge HR		Daily for duration of intervention		NR		NR		Participants met with study team to sync Fitbit weekly and problem-solve Fitbit-related issues		N/A			Godin Leisure-Time Exercise Questionnaire		
		Meints et al, 2019 [79]	Flex		Duration of intervention		Minimum of 10 h/day and 4 days/week		18 (out of 26) average valid weeks of Fitbit wear		Participants earned monetary reward for accurate use of Fitbit during first 2 weeks		N/A			NR		
		Painter et al, 2017 [80]	NR		Daily use		NR		NR		NR		NR			NR		
		Reed et al, 2019 [81]	Charge 2		Daily during waking hours		NR		NR		Basic instruction on using and setting up Fitbit		Yes; same use			Godin Leisure-Time Exercise Questionnaire		
		Wang et al, 2015 [82]	One		Duration of intervention		Minimum of 10 h/day		Nontypical days (not meeting wear time criteria) ranged from 5%-9%		NR		Yes			Accelerometry		
		Willis et al, 2017 [83]	Flex		Daily		NR		NR		NR		Yes			Accelerometry and self-reporting		
**Older adulthood (≥65 years)**																		
	**Intervention only**																	
		Ashe et al, 2015 [84]	One		Daily for 26 weeks		NR		NR		NR		No			Accelerometry		
		Christiansen et al, 2020 [85]	Zip		Daily during waking hours		NR		60% of intervention group monitored steps at least 80% of study time		In-person instruction of Fitbit use		No			Accelerometry		
		Kenfield et al, 2019 [86]	One		Duration of intervention		NR		Fitbits worn 98% of days during intervention		NR		No			Accelerometry and self-reporting		
		Thompson et al, 2014 [21]	NR		Daily for 48 weeks		NR		NR		NR		Yes; same use			Accelerometry		
	**Measurement only**																	
		Rossi et al, 2018 [87]	Alta		At all times for 30 days; remove only for bathing and sleeping		NR		Participants wore median of 93% of 30 days		Staff called participants after 1 week		N/A			Godin Leisure-Time Exercise Questionnaire		
		Schmidt et al, 2018 [88]	Charge HR		14 consecutive days during waking hours		NR		2 participants excluded for not wearing the device for a week		3 home visits		N/A			NR		
		Streber et al, 2017 [89]	Zip		During waking hours for 7 consecutive days		Minimum of 8 h/day		NR		No charging and no turning off and on		Yes; same use			Self-reporting		
	**Intervention and measurement**																	
		Harkins et al, 2017 [90]	Ultra		Daily		NR		NR		Daily email or text message and financial incentives for meeting goal		Yes; same use			Self-reporting		
		McMahon et al, 2017 [91]	One		During waking hours for 7 consecutive days		NR		Average hours worn at baseline: 13.01 (SD 1.87)		Participants asked to document days or times monitor was used; staff reviewed documentation and data		Yes; same use			Community Health Activities Model Program for Seniors Questionnaire		
		Vidoni et al, 2016 [92]	Zip		During waking hours		NR		NR		Staff made biweekly phone calls and additional calls if no activity for 3 days		Yes; device masked for 8 weeks versus 1 week			6-min walk test, mini-physical performance test, and battery of timed physical tasks		

^a^PA: physical activity.

^b^Only the reported intervention condition data.

^c^NR: not reported.

^d^N/A: not applicable.

^e^IPAQ: International Physical Activity Questionnaire.

### Childhood (9-12 Years)

#### General Study Characteristics

The 6 childhood studies had sample sizes ranging from 6 to 116 participants and were either single-group (n=3) or quasi-experimental designs (n=3). All studies were conducted in a school setting, and when appropriate, tried to integrate the intervention sessions into regular, daily school activities, including class sessions and recess periods. The most commonly used behavior change techniques were goal setting (through individual and group challenges) and positive reinforcement (through rewards). The duration of the intervention ranged between 4 and 12 weeks.

#### Fitbit Use

The most commonly used Fitbit model was the Fitbit Charge, which was used in 4 of the 6 interventions [18,23,25,26]. A total of 3 studies used Fitbits for both intervention and measurement purposes, 2 for intervention only, and 1 for measurement only. Participants in the comparison condition used Fitbit devices in only one of the 3 quasi-experimental studies.

#### Wear Time and Adherence

In total, 5 of the 6 interventions instructed participants to wear the device for a specific period. A total of 2 studies restricted device wear time to in-school supervised intervention sessions and reported that 100% of participants adhered to the device wear protocol, largely because of study staff monitoring [24,27]. The 2 interventions instructed participants to wear their Fitbits only during school days for the duration of the intervention [18,26]. In one study, participants were asked to wear the device for 24 hours during a 7-day period [25]. Applying a wear time criterion of 8 hours per day, one study reported that participants were adherent on 65%-73% of intervention days [23].

### Adolescence (13-17 Years)

#### General Study Characteristics

The 11 adolescent studies had sample sizes ranging from 6 to 388 participants. In total, 6 of the interventions used a randomized controlled trial design, 3 were quasi-experimental, and 2 used a single-group design. In total, 4 studies used an electronic or web-based platform for intervention delivery, including 3 that used mobile apps for data collection and the delivery of intervention content [28,29,33,36] and 1 that used Facebook as a web-based platform to encourage interactions between participants [30]. A total of 7 studies were delivered in a school setting [19,25,31-34,36]. Across all studies, the most commonly used behavioral change techniques were goal setting, self-monitoring, and knowledge shaping. The study duration varied between 4 weeks and 24 months.

#### Fitbit Use

The most commonly used Fitbit model was the Fitbit Flex, which was used in 5 of the 12 interventions [28,30,33,34,36]. The Fitbit Zip was the second most commonly used device (in 3 studies [31,32,35]). A total of 5 studies used Fitbits for both intervention and measurement purposes, 3 for intervention only, and 3 for measurement only. In 7 of the 10 studies with multiple conditions, participants in the comparison condition used Fitbit devices.

#### Wear Time and Adherence

Overall, 5 studies instructed participants to wear the device daily for the entire duration of the study [19,28,30,33,34], 4 studies instructed participants to wear the device for 7-day data collection periods only [25,32,35,36], and the remaining 2 studies did not report wear instructions [29,31,48]. Moreover, 5 studies used a minimum wear time criterion that was defined by either the number of hours (eg, 8 hours, 10 hours, or 24 hours per day) or steps (eg, 500 or 1000 steps per day) [19,30,32,35,36]. In addition, 3 studies reported the percentage of intervention days on which a specific minimum wear criterion was met (67.3% [19], 71.5% [30], and 73.4% [36]). One study excluded participants from the analysis who did not meet the wear time criterion [32]. One intervention that did not use the minimum wear time criterion was able to report an average number of days of valid Fitbit wear of 78.1 (SD 8.6; of a maximum of 84 days) for intervention participants [34]. Another study without a minimum wear time criterion reported that 36% of participants never wore their Fitbit [33].

#### Strategies to Boost Wear Time

Strategies to boost wear time included providing participants with oral and written instructions for Fitbit use [19,32]. Some studies also sent participants daily or weekly text messages or emails to encourage consistent use, meeting PA goals, or data upload [28-30,32]. In one study, a weekly lottery was used to reward participants with gift cards [33].

#### Other Measures of PA

Furthermore, 3 studies assessed PA with accelerometers at data collection time points [19,30,34], and 3 studies used self-report measures of PA [19,25,28].

### Early Adulthood (18-40 Years)

#### General Study Characteristics

The 20 eligible studies for adults aged 18-40 years had a range of sample sizes of participants. Randomized controlled trials (RCTs) were the most commonly used study design (11/20, 55% studies), followed by single-group study designs (5/20, 25% studies). In total, 12 of the 20 studies used mobile apps, web-based platforms, emails, or text messages for intervention delivery [41-44,46-51,55,56]. Of these studies, 3 encouraged web-based interactions between participants [41,44,47]. In total, 8 of the 20 studies used a campus- or workplace-based approach to intervention delivery [37,39,40,50-53,55]. Strategies for behavioral change included competition or challenges, both at the individual and group levels, and self-monitoring, social support, and goal setting. The study duration ranged from 1 week to 12 months.

#### Fitbit Use

The most commonly used Fitbit models were Fitbit Zip and Flex, which were used in 11 of the 20 studies [37,39,40,42,45,47,48,50,52,55,56]. Furthermore, 10 studies used Fitbits for both intervention and measurement purposes, 4 for intervention only, and 5 for measurement only. In 6 of the 15 studies with multiple conditions, participants in the comparison condition used Fitbit devices.

#### Wear Time and Adherence

All but 3 studies [37,41,44] instructed participants to wear the device daily, either at all times or during waking hours, for the duration of the intervention. Furthermore, 2 studies instructed participants to wear the device for a specific data collection period [41,44]. Different metrics were used to report adherence to daily wear instructions. There were 3 studies that reported the percentage of intervention days in which participants were adherent: 66% [45], 73% [44], and 78%-99% [47]. Another study reported that, on average, participants were adherent on 23.7 (SD 15.2) days (of 63 days) [55]. One study instructed participants to wear the device only during intervention sessions, and 100% of the participants were adherent [43]. Minimum wear time criteria were also used to report adherence. One study with a minimum wear time criterion of 1000 steps per day reported that participants met the criterion on 78% of intervention days [46], whereas another study in which the minimum wear time criterion was set at 500 steps per day reported that participants met the criterion on 60%-70% of intervention days [53]. A minimum criterion of 100 steps per day allowed one study to report a median number of 27.5 days (of 30) on which participants were adherent [52]. Another study with the same minimum wear time criterion reported that 51%-83% of participants were adherent [49]. With a minimum wear criterion of one day per week, one study reported that participants were adherent on 93% of intervention weeks on average [38].

#### Strategies to Boost Wear Time

Strategies to boost wear time included sending daily emails to inquire about Fitbit use experience [41], prompting participants to enter daily Fitbit data into an app [46], asking participants to share Fitbit data publicly [49], or sending daily reminder messages and instructions on Fitbit use [47]. Some studies provided participants with opportunities to win incentives based on compliance rates [53,54].

#### Other Measures of PA

A total of 10 studies asked participants to self-report their PA using instruments such as the International PA Questionnaire, the Stanford Brief PA Survey, and the 30-day PA Recall [37,39,40,42,44-46,50-52,54-56]. Only 1 study used an additional objective measure of PA (ie, accelerometer [38]).

### Middle Adulthood (41-64 Years)

#### General Study Characteristics

The sample sizes in the 28 middle adulthood studies ranged from 11 to 2113 participants. Most of the studies were RCTs (17/28, 61%), and 20 interventions used technology (eg, texts, apps, and social media) for intervention delivery [57,58,61,63,64,66,67,70-77,80-83,93]. The most common behavior change techniques used were self-monitoring, social support, behavioral counseling, and goal setting. The study duration ranged from 4 weeks to 6 months.

#### Fitbit Use

The most commonly used device was the Fitbit Flex, which was used in 9 studies [20,58,66,67,69,71,72,79,83]. There were 14 studies that used Fitbit for both intervention and measurement purposes, 11 for intervention only, and 3 for measurement only. Of the 18 studies with multiple conditions, 13 provided participants in the comparison condition with Fitbit devices.

#### Wear Time and Adherence

All but 3 studies [61,62,65] instructed participants to wear the device daily, either at all times or during waking hours, for the duration of the intervention. Among them, 2 studies reported the percentage of participants who were adherent to daily wear instructions: 96% [57] and 70% [20]. Other studies reported the percentage of days on which participants were adherent to wear instructions: 86% [58], 88% [66], 97% [71], 93% [72], and 76%-86% [64]. Furthermore, 9 studies also used a minimum wear time criterion defined by either the number of hours (eg, 8 or 10 hours per day) or steps (eg, 100 or 2000 steps per day) [59,62,67,69,71,74,75,79,82]. With a minimum wear time criterion of 100 steps per day, 1 study reported that 97% of the participants were adherent [69]. A minimum wear criterion of 10 hours per day allowed another study to report 18 of 26 average valid weeks of Fitbit wear [79], whereas another study used the same criterion to report that participants were adherent to the criterion on 6 days per week on average [75]. A minimum criterion of 10 hours per day was also used in another study to report 5%-9% of days on which participants did not meet the criterion on average [82]. Similarly, with a minimum wear time criterion of 1000 steps per day, another study reported 10.1%-12.7% of missing observation days [67]. Allowing participants to self-monitor PA as desired, one study reported the average hours worn of 17.3 (SD 5.7) hours per 6.1 (SD 0.8) days per week [65]. Another study excluded 3 participants who provided no Fitbit data [73].

#### Strategies to Boost Wear Time

Various strategies were used to promote Fitbit wear, including weekly texts to encourage PA based on Fitbit data [20], weekly emails providing activities’ progress summaries [64], asking participants to sync Fitbit data daily [68], providing incentives for wearing Fitbit regularly [70], public display of Fitbit data [58,71], and instructions on device use [71,73].

#### Other Measures of PA

Objective measures to assess PA were used in 12 studies [57-63,65,66,75,82,83], whereas self-reported measures were used in 11 studies [20,57,61,63,65,70,73,74,78,81,83].

### Older Adulthood

#### General Study Characteristics

The 10 older adulthood studies had sample sizes ranging from 25 to 102 participants, and most (8/10, 80%) were RCTs. Studies with older adults used individual and group-based approaches for intervention delivery. In addition to encouraging individualized PA goal setting or prescribing exercises, 3 studies involved regular phone calls made by study counselors or coaches [21,85,92]. One study provided participants with access to a study website and used text messages for intervention delivery [86]. Interventions providing PA education were often delivered in a group setting through a community-based approach, which allowed for the use of social support as a behavioral change technique [84,89,91]. Other behavioral change techniques included goal setting, behavioral counseling, and self-monitoring.

#### Fitbit Use

Different Fitbit devices were used across studies, including Classic, Zip, Ultra, Charge HR, and One, with none being predominant. In addition, 3 studies used Fitbit for both intervention and measurement purposes, 4 for intervention only, and 3 for measurement only. Of the 8 studies with multiple conditions, 5 provided participants in the comparison condition with Fitbit devices.

#### Wear Time and Adherence

All but 2 studies [89,91] instructed participants to wear the device daily, either at all times or during waking hours, for the duration of the intervention. Using daily wear instructions, the number of days the device worn was commonly reported either as an average (6.6, SD 1.1 over 7 days) [91] or as a median (93% over 30 days) [94]. One study reported that 60% of participants in the intervention group used Fitbit at least 80% of the study time [85], whereas another study simply reported that Fitbit was worn on 98% of days during the intervention [86]. One study used a minimum wear time criterion (8 hours per day) but did not report adherence to the criterion [89]. One study excluded 2 participants who did not wear the device for at least half of the instructed wear period (14 days) [88].

#### Strategies to Boost Wear Time

Strategies used to promote wear time adherence included providing participants with wear instructions and reminders via phone calls and text messages [85,87,90,92]. Some studies also asked participants to upload PA data on a daily basis or to document the device wear time and day [89,91].

#### Other Measures of PA

All but one study [88] used an additional measure of PA. Although self-reporting (using different scales) was the most common measure, which was used in 6 studies [86,89-92,94], accelerometers were used in 4 studies [21,84-86]. One study used a physical performance test along with a walk test [92].

## Discussion

### Principal Findings

This study reviewed the use of Fitbit devices in PA intervention studies across the life course. In addition to differences in study designs and intervention delivery methods, our results indicate considerable heterogeneity in Fitbit use within and between developmental stages. From early to older adulthood, most studies instructed participants to wear their Fitbit daily, either at all times or during waking hours, for the duration of the intervention. Studies conducted among children and adolescents tended to specify more limited device wear periods (eg, 24 hours for 7 days). Within developmental stages, our findings also suggest a lack of consistency in the definition of wear time criteria, which sometimes were used to report different adherence metrics or to exclude incomplete data from study analyses. A total of 8 different types of Fitbit devices were used across all age groups, with Fitbit Flex and Zip being the most predominant and some seemingly discontinuing use as newer devices became available. Regardless of intended Fitbit use (ie, measurement vs intervention tool), strategies to boost wear time were similar across stages, and the most commonly used strategies included sending participants reminders through texts or emails and asking participants to log their steps or sync their Fitbit data daily. Overall, the heterogeneity in Fitbit use across PA intervention studies reflects its relative novelty in the field of research.

Across all stages, based on the taxonomy developed by Lyons et al [95], the most common behavior change techniques used were self-monitoring and goal setting, regardless of the intended device use. This aligns with previous findings indicating goal setting and self-monitoring as the most commonly used behavior change techniques in studies with activity trackers [96]. As a self-monitoring technology, Fitbit devices provide real-time feedback that has the potential to stimulate behavior change. Self-monitoring allows participants to establish and track goals that were commonly operationalized through individual or group step count challenges. For example, a classroom-based study in children used individual step goals consistent with achieving 60 minutes of moderate-to-vigorous physical activity (MVPA) per day [23]. Additional behavioral change techniques appeared to be developmentally targeted. For example, among children, rewards for meeting step goals were often provided (eg, accruing points toward gift card balance). Through the use of social media platforms, adolescents and adults were provided with performance-based, web-based badges [41,97]. Among older adults, group-based PA education along with individual PA coaching or counseling provided social support to encourage the initiation and maintenance of behavior change [89].

Similar to behavior change techniques, the heterogeneity we observed regarding wear instructions and criteria also seemed to be because of developmental considerations. Most studies conducted among children and adolescents opted for instructions that required the device to be worn daily (8-24 hours) for a set data collection period (5-14 days); these studies did not set specific wear time criteria for inclusion in the analyses. Our findings align with previous results indicating a considerable reduction in the use of wearable trackers in youth following the first 2 weeks [19,98]. As such, limited device wear time in children and adolescents could potentially be a strategy that aims at capitalizing on wear patterns and usability trends in these groups. Studies conducted during early and middle adulthood tended to specify a minimum wear time criterion for inclusion in analyses based on specific numbers of steps or hours, in addition to daily wear instructions. However, studies conducted in older adults did not set minimum wear time criteria and instructed participants to wear the device daily during waking hours. The less rigid guidelines for device wear adherence among older adults could potentially be a way of increasing feasibility in populations who are less able to meet strict criteria and are less proficient in the use of technology [99].

Despite the importance of meeting a minimum threshold of wear time criteria to calculate a reliable estimate of PA, the results from this review also indicated a lack of consistency in the criteria used to define adherence to device wear *within* developmental stages. A systematic review that examined the length of device wear time required in PA interventions found that most studies conducted among adults did not report minimum device wear and that there was significant variation among studies reporting these criteria [22]. Corresponding to the lack of uniformity in wear time criteria, different metrics (eg, percentage, mean, and median) were used to report rates of adherence to wear instructions. If not met, the wear time criterion was sometimes used to exclude participants from the data analysis. However, many studies used the wear time criteria to report different metrics of adherence. Overall, the absence of clear reporting with standardized metrics significantly impaired efforts to assess overall adherence rates within developmental stages.

The most common pattern that emerged across studies was the use of reminder strategies to boost wear time, which did not differ by the intended device use (ie, intervention or measurement). Generally, texts and emails were sent on a daily or weekly basis as PA and Fitbit wear reminders. Manually logging or syncing Fitbit data on a daily basis was also a strategy to indirectly promote Fitbit wear on a daily basis. Results from previous studies indicate that, in addition to forgetting to wear their trackers [100], approximately 2% of study participants stopped using their devices each week altogether [101], and study participants also reported using their Fitbit less than 10% of the time following the end of wear-based incentives [102]. Therefore, these strategies are particularly essential given the evidence regarding decrease in Fitbit wear adherence over time in users and the need for reminder strategies to boost wear time [103].

Despite questions regarding the validity of Fitbits for assessing PA [104], most interventions in this review used Fitbit devices for both intervention and measurement purposes (39/75, 52%) or for data measurement purposes exclusively (15/75, 20%). Most studies (45/75, 63%) that were reviewed supplemented the use of Fitbit with additional objective (eg, accelerometers) or self-reported (eg, International PA Questionnaire) measures of PA. It is possible that the addition of other PA measures, even in studies that used Fitbit devices primarily as a measurement or data collection tool, was because of concerns about the uncertainty around the accuracy of measures provided by Fitbit devices [104]. In addition, the use of other measures (ie, accelerometry or self-reporting) to collect baseline or habitual activity [48] could also point to the perceived inaccuracy of data collected from commercially available trackers, which could have a potential impact on activity. Previous studies have also shown that commercially available trackers such as Fitbit devices often overestimate the time spent in MVPA compared with research-grade monitors [15,104,105].

However, the use of additional PA measures is not limited to addressing the accuracy issues. Results from a recent systematic review and meta-analysis of Fitbit-based interventions highlighted that the use of accelerometers and self-report, in addition to Fitbit, is often done to capture PA outcomes other than steps [106]. With the expansion of the use of Fitbit devices in PA intervention studies, previous studies have raised issues regarding their inability to capture PA constructs such as nonambulatory activities or energy expenditure [107]. In a recently published paper, Balbim et al [108] summarized the challenges and possible solutions to use Fitbit devices in mobile health intervention research. They described challenges and solutions at four different study phases: preparation, intervention delivery, data collection and analysis, and study closeout. For example, during the data collection phase, they point to the inaccuracy or unavailability of wear time data through Fitbit’s web API. They then discussed the potential solution of using heart rate data and pre-established rules for determining wear time and manually identifying gaps in heart rate data, indicating nonwear time. They also highlight the tedious and challenging nature of such an endeavor [108]. Thus, the use of additional PA measures (objective and subjective), despite increased burden on participants, allows for the efficient collection of different types of data, including valid wear time, information about body positions, sedentary behaviors, postural allocation, and the type of activity being performed [107,109-111].

### Strengths and Limitations

The primary limitation of this review is that the search for articles was restricted to articles available in the Fitabase library between 2012 and 2018 or on PubMed between 2019 and 2020. Given that the Fitabase library uses the systematic searching procedures of several databases (eg, PubMed, Google Scholar, and Science Direct), searching only PubMed for articles from 2019 to 2020 could have resulted in missed literature. In addition, this review was limited to intervention studies published in English and likely missed formative work that could provide important information regarding the design of Fitbit-based studies. Despite these limitations, this review provides insight into the current state of affairs in Fitbit use in research by focusing on different developmental stages and how the use of the device differs across those stages. Describing both study characteristics and the use of Fitbit devices provides insight into PA study designs across the lifespan and the different ways in which these monitoring devices are used.

### Conclusions

Insufficient PA across the lifespan is associated with an increased risk of numerous chronic diseases and is a major public health issue [1]. The prominence and relatively low cost of Fitbit devices have increased their use by the public and researchers as PA trackers. Although behavior change techniques and strategies to boost Fitbit wear time were similar across all studies reviewed, our findings indicate significant differences in wear instructions and metrics for reporting adherence rates. Although between-stage differences appear to be based on developmental considerations that aim to maximize device use in each age group, within-group differences appear to result from a lack of uniformity in metrics used to report rates of adherence and minimum wear time criteria. The use of additional PA data collection tools in most studies that were reviewed points to the accuracy issues raised by previous research focusing on Fitbits in PA interventions [104,105] and a reluctance to rely on Fitbits as the primary measurement device or for the assessment of habitual activity. However, additional PA measures are also used to capture PA constructs not measured by Fitbit devices (eg, MVPA, sedentary behaviors, and types of activity). As the use of monitoring devices continues to expand in the field of PA research, the lack of uniformity in study protocols and metrics of reported measures represents a major issue for purposes of comparison [112]. Given that clinical trial registries serve as a repository for researchers [113], there is a need for increased transparency in the prospective registration of PA intervention studies. This paper serves as a call for researchers using Fitbit devices to provide a clear rationale for the use of several PA measures and to specify the metrics that will be reported for each. By providing researchers with a synthesis of information on the use of Fitbit devices in PA intervention studies across the life course, this narrative review serves as a resource that may be used to inform the design of future trials involving Fitbit devices.

## Abbreviations

API: application programming interface
MVPA: moderate-to-vigorous physical activity
PA: physical activity
RCT: randomized controlled trial
